# Dose-Dependent Effects of Resveratrol on Cisplatin-Induced Hearing Loss

**DOI:** 10.3390/ijms22010113

**Published:** 2020-12-24

**Authors:** Chang Ho Lee, Kyung Woon Kim, So Min Lee, So Young Kim

**Affiliations:** Department of Otorhinolaryngology-Head & Neck Surgery, CHA University College of Medicine, Seongnam 13496, Korea; hearwell@gmail.com (C.H.L.); wgaltz@naver.com (K.W.K.); lws6812@naver.com (S.M.L.)

**Keywords:** hearing loss, cisplatin, resveratrol, nuclear factor kappa B, aryl hydrocarbon receptor

## Abstract

Previous preclinical studies have demonstrated the otoprotective effects of resveratrol (RV) at low doses. This study aimed to investigate the dose-dependent effects of RV in rats with cisplatin (CXP)-induced hearing loss. Sprague-Dawley rats (8-weeks old) were divided into six treatment groups (*n* = 12/group) and treated as follows: control, 0.5 mg/kg RV, 50 mg/kg RV, CXP, 0.5 mg/kg RV + CXP), and 50 mg/kg RV + CXP groups. CXP (3 mg/kg) was intraperitoneally injected for 5 days. RV (0.5 or 50 mg/kg) was intraperitoneally injected for 10 days from the first day of CXP administration. Auditory brainstem response (ABR) thresholds were measured before and within 3 days at the end of the drug administration. Cochlear tissues were harvested, and the outer hair cells were examined using cochlear whole mounts. The mRNA expression of *NFκB, IL6, IL1β*, and *CYP1A1*, and protein levels of aryl hydrocarbon receptor (AhR) and cytosolic and nuclear receptor for advanced glycation endproducts (RAGE) were evaluated. The ABR threshold increased in the 50 mg/kg RV and CXP groups at 4, 8, 16, and 32 kHz. The 0.5 mg/kg RV + CXP group demonstrated decreased hearing thresholds at 4 and 32 kHz compared to the CXP group. Cochlear whole-mount analysis revealed loss of outer hair cells in the 50 mg/kg RV and CXP groups and partial prevention of these cells in the 0.5 mg/kg RV + CXP group. The mRNA expressions of *NFκB, IL6*, and *IL1β* were increased in the 50 mg/kg RV and CXP groups compared to the control group. In contrast, these levels were decreased in the 0.5 mg/kg RV + CXP group compared to the CXP group. The mRNA expression of *CYP1A1* was increased in the CXP group, while it was decreased in the 0.5 mg/kg RV + CXP group compared to the control group. The protein levels of AhR and cytosolic RAGE decreased in the 0.5 mg/kg RV group. Low-dose RV had partial otoprotective effects on CXP ototoxicity. The otoprotective effects of RV may be mediated through anti-oxidative (*CYP1A1* and RAGE) and anti-inflammatory (*NFκB, IL6,* and *IL1β*) responses. High-dose RV exerted an inflammatory response and did not ameliorate CXP-induced ototoxicity.

## 1. Introduction

Resveratrol (*trans*-3,4′,5,-trihydroxystilbene, RV) is a natural polyphenol abundant in grape skin [1]. RV is the key compound responsible for the “French paradox”, which refers to a reduced risk of coronary heart disease in the French population, despite its high-saturated-fat diet [2]. A moderate dose of RV has been reported to have protective effects on cardiovascular diseases through anti-oxidative effects, mediated by scavenging peroxyl radicals and impeding lipid peroxidation, and anti-inflammatory effects, via several target molecules, including nuclear factor kappa B (NFκB) [3]. Additionally, increasing evidence supports the anti-proliferative and anti-angiogenic effects of RV, and RV-mediated effects have been implicated in multiple conditions, from cardiovascular and neurodegenerative diseases to cancer and longevity [4].

The effects of RV vary depending on its bioavailability and dose. The bioavailability of RV is dependent on the administration route and is affected by the food matrix. When administered orally, the sulfated form is the main metabolite in human plasma and limits the bioavailability of RV [5]. The absorptive efficiency is influenced by the constituents, and only about 1.7–1.9% of orally administered resveratrol was accounted for as biologically active free polyphenol [6]. In in vitro studies, a high dose of RV (10^−5^–10^−4^ M) has been tested for cancer chemoprevention effects, which is much higher than in the in vivo doses [7]. In addition, the dose-dependent effects of RV have been reported to include anti-apoptotic and cardioprotective effects at low doses and pro-apoptotic and vascular endothelial injuries at high doses [8].

A number of preclinical studies have reported the protective effects of RV in animals with hearing loss induced by aging [9,10,11], noise [12,13], aminoglycoside [14,15], and cisplatin [16,17,18]. The plausible mechanisms of these otoprotective effects of RV include amelioration of oxidative stress and inflammation [15] and restoration of autophagy [19]. However, a dose-dependent otoprotective effect of RV was suggested in a cisplatin-induced rat study [20]. The study showed a hearing-preservation effect of RV on cisplatin-administered rats at a low dose of 0.1 mg/kg/day of RV for 10 days, whereas enhanced ototoxicity was observed at high doses of 1 or 10 mg/kg/day of RV for 10 days [20]. Another noise-induced rat study demonstrated no significant hearing preservation effect with 30 mg/kg of RV in rats [21].

We hypothesized that there exists a dose range for the otoprotective effect of RV. To test this hypothesis, rats were administered low (0.5 mg/kg/day) or high (50 mg/kg/day) doses of RV based on a previous study that demonstrated no protective effect of RV at 50 mg/kg in the vascular endothelial function in rats [22].

## 2. Results

### 2.1. ABR Threshold Shift Following CXP and/or RV Administration

**Figure 1:**
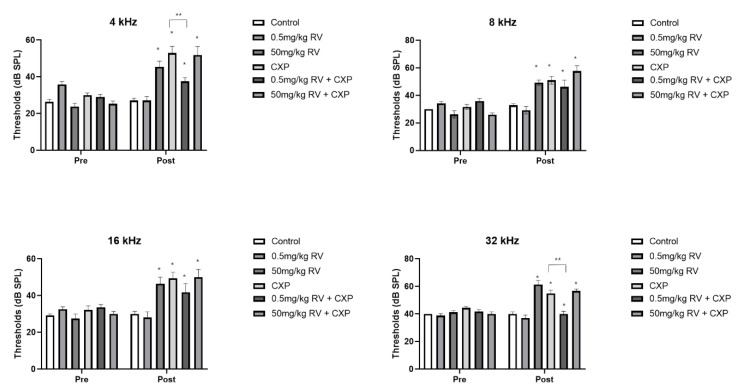
The auditory brainstem response thresholds of each group. The 50 mg/kg RV + CXP group demonstrated increased hearing thresholds that were not significantly different from those of the CXP group. In the 0.5 mg/kg RV + CXP group. the hearing thresholds after drug administration were lower than those of the CXP group at 4 and 32 kHz (* *p* < 0.05, pre- vs. post-treatment, ** *p* < 0.05, CXP vs. 0.5 mg/kg RV + CXP groups).

Among the six groups, four groups (other than control and 0.5 mg/kg RV groups) demonstrated ABR threshold shifts following drug administration (Figure 1). Although the control and 0.5 mg/kg RV groups did not show increased hearing thresholds after drug administration, the 50 mg/kg RV group demonstrated increased hearing threshold at 4, 8, 16, and 32 kHz (23.75 ± 1.83 vs. 45.45 ± 3.12 dB SPL, *p* < 0.001 for 4 kHz, 26.25 ± 2.63 vs. 49.29 ± 1.95 dB SPL, *p* < 0.001 for 8 kHz, 27.50 ± 2.50 vs. 46.43 ± 3.57 dB SPL, *p* = 0.001 for 16 kHz, and 41.25 ± 1.25 vs. 61.43 ± 2.94 dB SPL, *p* = 0.02 for 32 kHz). Cisplatin administration induced elevation of the hearing threshold. The CXP group demonstrated increased hearing threshold after CXP injection at 4, 8, 16, and 32 kHz (30.00 ± 1.21 vs. 52.94 ± 3.61 dB SPL, *p* < 0.001 for 4 kHz, 31.67 ± 1.85 vs. 51.11 ± 2.79 dB SPL, *p* < 0.001 for 8 kHz, 32.22 ± 2.22 vs. 49.44 ± 3.28 dB SPL, *p* < 0.001 for 16 kHz, and 44.44 ± 1.21 vs. 55.00 ± 2.32 dB SPL, *p* < 0.001 for 32 kHz). The 50 mg/kg RV + CXP group demonstrated increased hearing threshold after CXP injection at 4, 8, 16, and 32 kHz, and the hearing thresholds after drug administration (post) were not significantly different from those of the CXP group (25.39 ± 1.44 vs. 51.82 ± 4.64 dB SPL, *p* < 0.001 for 4 kHz, 26.00 ± 1.31 vs. 57.69 ± 3.95 dB SPL, *p* < 0.001 for 8 kHz, 30.00 ± 1.48 vs. 50.00 ± 4.26 dB SPL, *p* < 0.001 for 16 kHz, and 40.00 ± 1.48 vs. 56.67 ± 1.42 dB SPL, *p* < 0.001 for 32 kHz). The 0.5 mg/kg RV + CXP group also demonstrated increased hearing threshold after CXP injection at 4, 8, 16, and 32 kHz, but the hearing thresholds after drug administration (post) were lower than those of the CXP group at 4 and 32 kHz (29.09 ± 1.46 vs. 37.50 ± 2.14 dB SPL, *p* = 0.008 (*p* = 0.02, vs. CXP group, unpaired *t*-test) for 4 kHz, 35.91 ± 1.82 vs. 46.25 ± 4.91 dB SPL, *p* = 0.034 for 8 kHz, 33.64 ± 1.55 vs. 41.88 ± 4.76 dB SPL, *p* = 0.071 for 16 kHz, and 41.82 ± 1.26 vs. 40.00 ± 2.04 dB SPL, *p* = 0.576 (*p* = 0.05, vs. CXP group, unpaired *t*-test) for 32 kHz).

### 2.2. Cochlear Outer Hair Cell Injury

**Figure 2:**
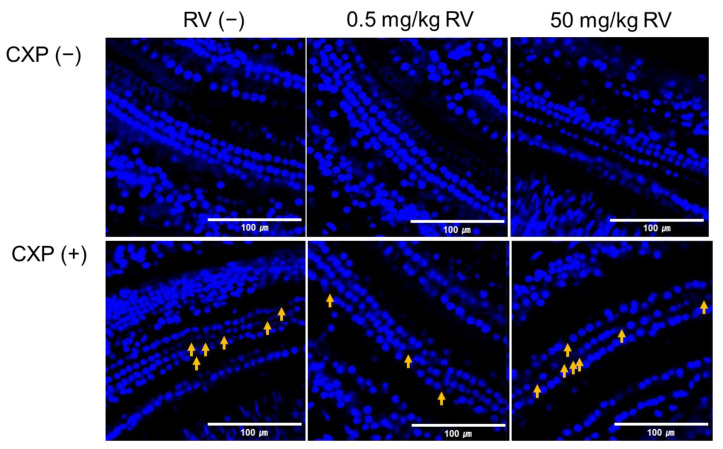
The loss of outer hair cells were noted in the 50 mg/kg RV group. (yellow arrow: loss of outer hair cells).

Cochlear whole-mount examination revealed a loss of outer hair cells in the 50 mg/kg RV group (Figure 2). CXP administration caused disorientation and reduction of the outer hair cells. These outer hair cell injuries were attenuated in the 0.5 mg/kg RV + CXP group but not in the 50 mg/kg RV + CXP group.

### 2.3. Increased NFκB, IL1β, and IL6 and Decreased CYP1A1 Levels in CXP-Induced Hearing Loss Rats and Partial Reversal of Expression Patterns Following 0.5 mg/kg RV Treatment

**Figure 3:**
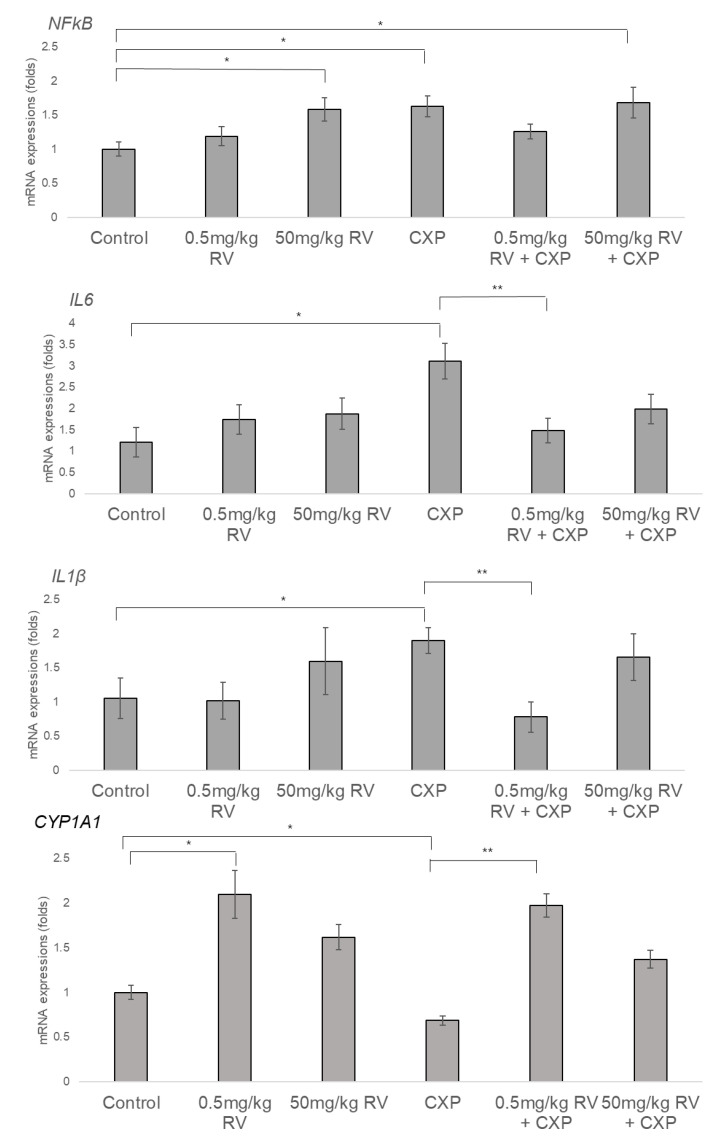
Compared to the CXP group, the 0.5 mg/kg RV + CXP group showed decreased mRNA levels of *NFκB, IL1β*, and *IL6*. The 50 mg/kg RV + CXP group did not show any significant difference in mRNA levels of *NFκB, IL1β*, and *IL6* compared to the CXP group. The mRNA level of *CYP1A1* was increased in the 0.5 mg/kg RV group and 0.5 mg/kg RV + CXP group. (* *p* < 0.05, compared to control group, ** *p* < 0.05, compared to CXP group).

The CXP group showed significantly higher mRNA levels of *NFκB, IL1β*, and *IL6* (1.63 ± 0.14 (*p* = 0.001)*,* 2.22 ± 0.39 (*p* = 0.03), and 2.25 ± 0.24 (*p* = 0.02) fold change, respectively) compared to the control group (Figure 3). The 50 mg/kg RV group and the 50 mg/kg RV + CXP groups showed higher mRNA levels of *NFκB* (1.58 ± 0.16 fold, *p* = 0.005 for 50 mg/kg RV group and 1.68 ± 0.20 fold, *p* = 0.007 for 50 mg/kg RV + CXP group) compared to the control group. The 0.5 mg/kg RV group and 0.5 mg/kg RV + CXP group did not show a significant change in mRNA levels of *NFκB, IL1β*, and *IL6*, compared to the control group.

Compared to the CXP group, the 0.5 mg/kg RV + CXP group showed decreased mRNA levels of *NFκB*, *IL1β*, and *IL6* (1.63 ± 0.14 vs. 1.26 ± 0.10, *p* = 0.04 for *NFκB*, 2.22 ± 0.39 vs. 0.78 ± 0.22, *p* = 0.006 for *IL1β* and 2.25 ± 0.24 vs. 1.35 ± 0.24, *p* = 0.018 for *IL6*). The 50 mg/kg RV + CXP group did not show any significant difference in mRNA levels of *NFκB, IL1β*, and *IL6* compared to the CXP group.

The mRNA expression level of *CYP1A1* was increased in the 0.5 mg/kg RV group but decreased in the CXP group compared to the control group (2.1 ± 0.27 vs. 1.0 ± 0.08, *p* = 0.003 for the 0.5 mg/kg RV group and 0.68 ± 0.05. vs. 1.0 ± 0.08, *p* = 0.006 for CXP group). *CYP1A1* mRNA level, which was decreased in the CXP group, was increased in the 0.5 mg/kg RV + CXP group but not in the 50 mg/kg RV + CXP group (2.0 ± 0.13 vs. 0.68 ± 0.05, *p* < 0.001).

### 2.4. Decreased AhR Protein and Nuclear/Cytosolic RAGE Levels Following RV Administration

**Figure 4:**
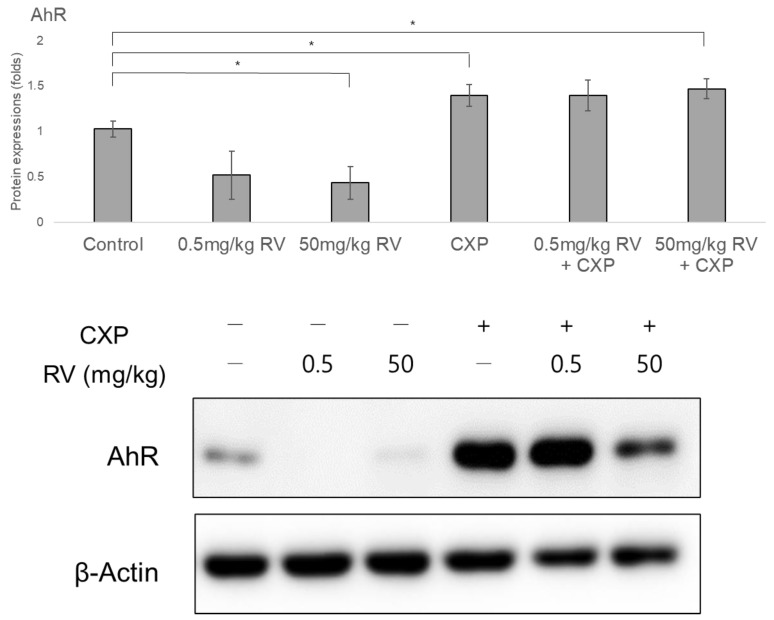
The administration of RV decreased the AhR protein levels. On the other hand, CXP administration increased the AhR protein level (* *p* < 0.05, compared to the control group).

AhR protein levels decreased following administration of a high dose of RV (Figure 4). The 50 mg/kg RV group showed lower AhR protein level compared to the control group (0.44 ± 0.17 fold, *p* = 0.006). CXP administration increased the AhR protein level in the CXP group and the 50 mg/kg RV + CXP groups (1.40 ± 0.11 fold, *p* = 0.019 for CXP group and 1.47 ± 0.10 fold, *p* = 0.003 for the 50 mg/kg RV + CXP group). AhR protein levels showed a decreased tendency in the 0.5 mg/kg RV group and an increased tendency in the 0.5 mg/kg RV + CXP group compared to the control group, although there was no statistical significance (0.52 ± 0.25 fold, *p* = 0.0.7 for the 0.5 mg/kg RV group and 1.40 ± 0.16 fold, *p* = 0.053 for the 0.5 mg/kg RV + CXP group).

**Figure 5:**
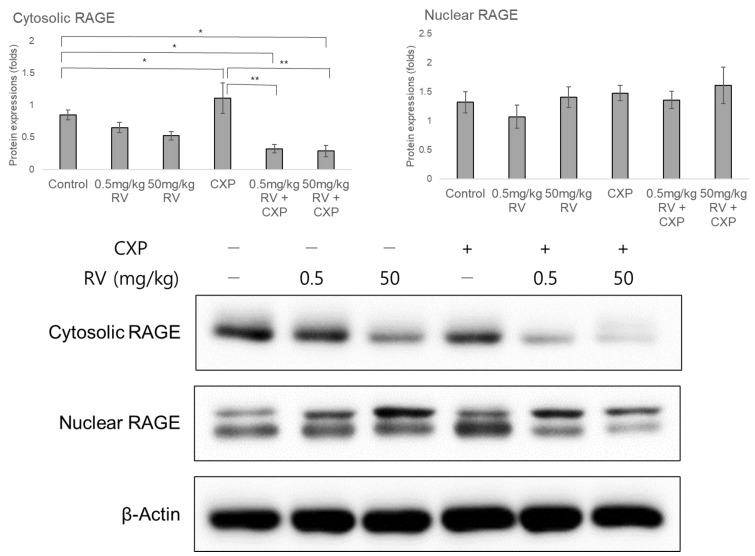
Cytosolic RAGE protein level decreased following administration of a 50 mg/kg of resveratrol (RV). Both 0.5 mg/kg RV + CXP and 50 mg/kg RV + CXP groups showed lower cytosolic RAGE protein levels compared to the control group. (* *p* < 0.05, compared to the control group, ** *p* < 0.05, compared to the CXP group)

Cytosolic RAGE protein level decreased following administration of a high dose of RV (Figure 5). The 50 mg/kg RV group showed significantly lower cytosolic RAGE protein level compared to the control group (0.32 ± 0.06 fold, *p* = 0.001). The CXP group did not show any significant difference in cytosolic RAGE protein level compared to the control group (1.11 ± 0.22 fold, *p* = 0.28). Both 0.5 mg/kg RV + CXP and 50 mg/kg RV + CXP groups showed lower cytosolic RAGE protein levels compared to the control group (0.60 ± 0.08 fold, *p* = 0.03 for the 0.5 mg/kg RV + CXP group and 0.47 ± 0.06 fold, *p* = 0.001 for the 50 mg/kg RV + CXP group). The nuclear RAGE protein levels were not different among the groups.

## 3. Discussion

In this study, we demonstrated that low-dose RV partially reduced the auditory threshold shifts in CXP-induced hearing loss rats. The increased expression of the pro-inflammatory cytokines IL1β, IL6, and cytosolic RAGE in CXP rats were attenuated in the low-dose RV + CXP rats. Low-dose RV elevated *CYP1A1* transcript and the decreased *CYP1A1* mRNA expression in CXP rats was reversed following co-administration of low-dose RV with CXP. In contrast, high-dose RV increased the auditory thresholds. High-dose RV did not attenuate the mRNA expression levels of pro-inflammatory cytokines. High-dose RV alone elevated the mRNA expression level of *NFκB*. Both low- and high-dose RV alone decreased AhR and cytosolic RAGE protein levels. Although they did not reduce AhR, cytosolic RAGE expression was decreased in the groups co-administered high or low doses of RV along with CXP. By investigating the dose-dependent otoprotective effects of RV on relevant molecules, this study contributes substantially to the existing body of knowledge in the field.

In this study, we found that low-dose RV reduced hearing loss and inflammatory responses. A few prior studies have reported the otoprotective effects of low to moderate doses of RV [9,10,13,15,17,18]. Long-term administration of RV reduced age-related hearing loss in mice by protecting the cochlear hair cells, spiral ganglion cells, and stria vascularis [9]. In the study, the authors demonstrated that these effects of RV were mediated through activation of SIRT1, which rebalanced mitochondrial biogenesis and mitophagy to overcome oxidative stress [9]. An in vitro study also demonstrated the anti-oxidative effects of RV mediated through activation of mitochondrial biogenesis in mouse cochlea and HEI-OC1 cells [10]. The anti-oxidative effects of RV (5 mg/kg) were also validated in noise-induced hearing loss rats that reduced reactive oxygen species (ROS) and cyclooxygenase 2 levels [13]. Aminoglycoside-induced hearing loss was also prevented by administration of RV (10 mg/kg) through the modulation of genes associated with oxidative (glutathione peroxidase 1, superoxide dismutase 1, copper chaperone for superoxide dismutase, and NADPH oxidase activator 1) and inflammatory responses (IL1β, IL4, myeloperoxidase, and neutrophil cytosolic factor 1) [15]. Reduced ROS and hearing preservation have been reported in cisplatin-induced hearing loss in guinea pigs and rats following administration of 10 mg/kg of RV [17,18]. In contrast, RV was shown to mediate ototoxic effects at high doses [20]. To the best of our knowledge, no prior study has investigated the ototoxic effect of RV. The results of our study imply that high doses of RV induce inflammatory responses associated with the expression of *NFκB, IL6*, and *IL1β*.

The hearing preservation effects of low-dose RV observed in this study may be mediated by the anti-inflammatory effects of *NFκB, IL6*, and *IL1β*, and anti-oxidative effects of *CYP1A1* and cytosolic RAGE. CXP increases oxidative stress and the pro-inflammatory cytokines *NFκB* and *IL6* [23]. RV has been reported to decrease RAGE and NFκB activities, thereby restoring oxidative stress and inflammation-related diseases [24]. In diabetic rats, RAGE expression levels with malondialdehyde level, total oxidant, and plasma glucose, but not AGE level, were decreased following RV treatment [25]. In mouse macrophages, AGE and lipopolysaccharides stimulated RAGE, which induced NF-κB activation and pro-inflammatory cascades including IL6 and IL1β, and RV reduced these inflammatory responses of RAGE/NF*κ*B/IL6 and IL1β [26]. In the present study, CXP rats showed increased levels of AhR expression. This result is consistent with a previous study that demonstrated the increased AhR in cisplatin-induced rats [27]. The increased AhR expression in CXP rats was reversed by low-dose or high-dose RV administration in the present study. In line with these results, the AhR activated by the endogenous ligand of tryptophan metabolite-derived indoxyl sulfate and the subsequent AhR-mediated endothelial hyperpermeability were reversed by RV in bovine aortic endothelial cells [28].

The high dose of RV did not show otoprotective effects in the present study. A few previous studies have suggested the adverse effects of RV overdose [8,29]. The pro-oxidant effects of a high dose of RV could mediate the ototoxic effects in this study. The high levels of pro-inflammatory molecules of *NFkB* and *IL6* and decrease of anti-oxidative molecule of *CYP1A1* may mediate the ototoxic response of a high dose of RV in this study. Previous studies reported the pro-oxidant effects of a high dose of RV in the cardiovascular system [30,31]. Because activation of *CYP1A1* was reported to be mediated by AhR, the suppression of AhR expression with a high dose of RV could be linked with the insufficient activation of *CYP1A1* [32]. In addition, the exaggerated apoptotic effects of RV at high doses could hinder the otoprotective effects in this study. Both intrinsic and extrinsic apoptotic pathways are activated by RV [33]. Mitochondrial membrane potential, Bax/Bcl-2 ratio, and cleaved caspase-8 and -3 were increased after RV administration [33]. In addition, unidentified metabolites of RV in vivo could impact the high-dose RV rats. In contrast to in vitro studies, the tissue concentrations of RV might be time-dependent, and pharmacokinetic-pharmacodynamic profiles, including the maximal concentration, threshold concentration, and total exposure over time, need to be considered in an in vivo study [34]. A previous study reported the ototoxic effect of resveratrol with a cumulative dose of 100 mg/kg (Table 1) [20]. However, another study reported an otoprotective effect of resveratrol with a cumulative dose of 100 mg/kg (Table 1) [35]. The cumulative dose up to 50 mg/kg of resveratrol might be otoprotective according to previous results [16,17,18,20]. The adequate dose of RV needs to be further evaluated in preclinical and clinical studies on the application of RV in hearing loss patients.

## 4. Materials and Methods

### 4.1. Experimental Design

**Figure 6:**
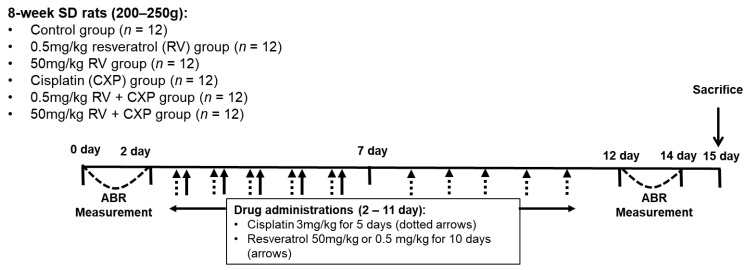
The experimental schedule of the present study. The cisplatin (CXP) group was administered CXP (3 mg/kg/day) for 5 days and then normal saline every day for 5 days. The RV + CXP groups were administered RV along with CXP every day for 5 days and then RV every day for 5 days.

The animal experiments performed in this study were approved by the Institutional Animal Care and Use Committee of the CHA University Medical School (IACUC200025, approval date: 20191206). All experimental procedures complied with the guidelines of the Institutional Animal Care and Use Committee of the CHA University Medical School. Seventy-two female Sprague-Dawley rats (8-weeks old) were divided into six groups (*n* = 12/group; Figure 6). The control group received 500 µL of normal saline (intraperitoneal injection (i.p.)) every day for 10 days. The 0.5 mg/kg RV group was administered 0.5 mg/kg of RV (i.p.) every day for 10 days. The 50 mg/kg RV group was administered 50 mg/kg of RV (i.p.) every day for 10 days. The cisplatin (CXP) group was administered CXP (3 mg/kg/day) (i.p.) for 5 days and then 500 µL of normal saline (i.p.) every day for 5 days. The 0.5 mg/kg RV + CXP group was administered 0.5 mg/kg of RV along with 3 mg/kg of CXP (i.p.) every day for 5 days and then 0.5 mg/kg of RV (i.p.) every day for 5 days. The 50 mg/kg RV + CXP group was administered 50 mg/kg of RV along with 3 mg/kg of CXP (i.p.) every day for 5 days and then 50 mg/kg of RV (i.p.) every day for 5 days. The auditory brainstem response (ABR) thresholds were measured before (0–2 days) and after (12–14 days) the drug administration schedule. All rats were sacrificed three days after the end of the drug administration schedule (15 days). No rats died during the experiments.

### 4.2. ABR Measurements

The ABR thresholds at 4, 8, 16, and 32 kHz were measured using the SmartEP system as described previously [23,24]. The rats were intraperitoneally administered zoletil (40 mg/kg) and xylazine (10 mg/kg) prior to the ABR measurements. The reference and ground electrodes were inserted at the vertex and contralateral thigh, and the measuring electrode was placed at the ipsilateral retroauricular area. Tone bursts of 4, 8, 16, and 32 kHz with durations of 1562 µs with Blackman envelope were applied at a stimulation rate of 21.2/s to the EC1 electrostatic speaker. The auditory-evoked responses of 1024 sweeps were averaged. The intensity of the sound stimuli was applied up to 90 dB SPL. The lowest sound intensity with wave III was defined as the ABR threshold.

### 4.3. Cochlear Whole Mounts

Outer hair cells were histologically examined using cochlear whole mounts. Two rats per group (total 24 ears for 12 rats) were used for cochlear whole mounts. The cochleae were fixed in 4% paraformaldehyde solution. After decalcification, the cochlear outer hair cells were dissected. Then, tissues were soaked in 0.3% Triton blocking solution. The 4′,6-diamidino-2-phenylindole dihydrochloride (DAPI) solution was applied to the tissues for 1 h, and the tissues were then mounted on slides and examined under a light microscope. The number and arrangement of cochlear outer hair cells were examined using confocal microscopy with a stack image under 400-fold magnification (Zeiss LSM 880, Zeiss, Oberkochen, Land Baden-Wurttemberg, Germany).

### 4.4. mRNA Expression of Inflammatory Factors

Five rats per group (a total of 60 ears of 30 rats) were used for quantitative reverse transcription-polymerase chain reaction (qRT-PCR). qRT-PCR was performed as described previously [25]. The membranous labyrinth tissues were collected and frozen in a NO_2_ deep freezer. Total RNA was extracted within 24 h of tissue collection using TRIzol™ Reagent (Invitrogen, Waltham, MA, USA). The purified RNA was checked for purity and quantity by measuring the 260/280 nm absorbance ratio using a Micro UV-Vis spectrophotometer (Lifereal Biotechnology Corp. Ltd., Hangzhou, China). Only samples with a 260/280 ratio > 1.8 and a 260/230 ratio > 1.5 were used for qRT-PCR. Maxime™ RT Pre Mix (Oligo (dT)15 Primer) (iNtRON Biotechnology, Seongnam, Korea) was used for reverse transcription. Nuclear factor-κB (*NFκB*), interleukin-1β (*IL1β*), interleukin-6 (*IL6*), and cytochrome P450 1A1 (*CYP1A1*) were reverse transcribed and PCR-amplified using the primers listed in Table 2. Real-time reverse transcription (RT)-PCR was performed on a ViiA7 Real-time PCR system (Applied Biosystems, Carlsbad, CA, USA) using TOPreal™ qPCR 2× PreMIX (SYBR Green with low ROX; Enzynomics, Daejeon, Korea) using the following protocol: initial activation of HotStarTaq^®^ DNA polymerase at 95 °C for 15 min, followed by 50 cycles of 95 °C for 10 s, 60 °C for 15 s, and 72 °C for 15 s. The amplification efficiency (E) of each amplicon was determined using a 10-fold serial dilution of positive control complementary DNA (cDNA) and calculated from the slopes of the log input amounts (20 ng–2 pg of cDNA) that were plotted according to the crossing point values using the formula E = 10 − 1/slope. All primer efficiencies were confirmed to be high (>90%) and comparable. The calculated mRNA levels were normalized to glyceraldehyde 3-phosphate dehydrogenase (GAPDH) according to the formula 2^−ΔΔCt^, and expressed as a percentage of the reference gene.

### 4.5. Protein Levels of Aryl Hydrocarbon Receptor (AhR) and Receptor for Advanced Glycation Endproducts (RAGE)

Five rats per group (a total of 60 ears of 30 rats) were used for Western blotting. Two rats per group (a total of 24 ears of 12 rats) were used to measure AhR protein level, and the remaining three rats in each group (total 36 ears of 18 rats) were used to measure protein levels of cytosolic/nuclear RAGE.

Nuclear and cytosolic fractions from cochlear tissue were extracted using the NE-PER Nuclear Cytoplasmic Extraction Reagent kit (Pierce, Rockford, IL, USA). Briefly, the tissue was washed twice with cold PBS and centrifuged at 500× *g* for 5 min. The pellet was resuspended in 400 μL of cytoplasmic extraction reagent I by homogenizing. The suspension was then incubated on ice for 10 min followed by the addition of 22 μL of cytoplasmic extraction reagent II. The mixture was vortexed for 5 s, incubated on ice for 1 min, and centrifuged for 5 min at 16,000× *g*. The supernatant (cytoplasmic extract) was transferred to a microcentrifuge tube. The insoluble pellet was resuspended in 200 μL of nuclear extraction reagent by vortexing for 15 s, incubated on ice for 10 min, and centrifuged for 10 min at 16,000× *g*. The resulting supernatant constituted the nuclear extract.

Radioimmunoprecipitation assay buffer (Cell Signaling Technology, Danvers, MA, USA) was used for tissue lysis. The protein concentration was evaluated using a Bio-Rad Protein Assay Kit. The proteins were separated using 8% sodium dodecyl sulfate-polyacrylamide gel electrophoresis. Gels were transferred to polyvinylidene difluoride membranes (Merck Millipore, Burlington, MA, USA) and soaked in blocking buffer (5% nonfat dry milk in Tris-buffered saline containing Tween-20 (TBS-T)) for 1 h. The membrane was incubated in primary antibodies against AhR (mouse monoclonal, Santa Cruz Biotechnology, #SC-133088), nuclear RAGE (ab3611, rabbit polyclonal, Abcam, Cambridge, MA, USA), cytosolic RAGE (MAB1179, Rat monoclonal, R&D system, Minneapolis, MN, USA), β-actin (D6A8, rabbit mAb; Cell Signaling Technology, Danvers, MA, USA), and HDAC1 (sc47778, mouse, Santa Cruz, Dallas, TX, USA). The membranes were then incubated with the corresponding horseradish peroxidase (HRP)-conjugated secondary antibodies (anti-rabbit IgG, HRP-linked; Cell Signaling Technology, #7074S and goat anti-mouse IgG H&L (HRP); Abcam, #ab97023). The protein bands were then visualized using an enhanced chemiluminescence kit (Bio-Rad, Hercules, CA, USA). Protein expression levels were calculated using ImageJ gel analysis software (National Institutes of Health, Bethesda, MD, USA) and compared with the expression levels of β-actin (for cytosolic proteins) or HDAC (for nuclear RAGE).

### 4.6. Statistical Analysis

Changes in ABR thresholds were analyzed using paired *t*-test within each group and unpaired *t*-test between groups. The differences in mRNA and protein levels were analyzed using unpaired *t*-tests. The values are presented as mean ± standard deviation. Statistical significance was defined as *p* < 0.05. All analyses were performed using SPSS 21.0 (IBM Corp., Armonk, NY, USA).

## 5. Conclusions

Low-dose RV partially preserved hearing in cisplatin-induced hearing loss rats. The increased anti-oxidative effects involving *CYP1A1* expression might be linked to the otoprotective effects of low-dose RV. The increased expression of pro-inflammatory cytokines *IL6* and *IL1β* in CXP rats was attenuated in the low-dose RV + CXP rats. However, high-dose of RV did not exert otoprotective effects in CXP-induced hearing loss rats.

## Figures and Tables

**Table 1:** Studies on the effects of resveratrol on the cisplatin-induced ototoxicity. i.p., intraperitoneal injection; N/A means not applicable.

Animals	Dose of Resveratrol (mg/kg)	Cumulative Dose (mg/kg)	Dose of Cisplatin (mg/kg)	Ototoxic/Otoprotective	References
SD 8-weeks rat	0.5/day for 10 days (i.p.)	5	3 for 5 days	Otoprotective	Current study
Wistar albino rat	0.1, 1/day for 10 days (i.p.)	1, 10	16	Otoprotective	Olgun Y. et al., 2014 [20]
Adult albino guinea pig	10/day for 2 days (i.p.)	20	10 for 1 day	Otoprotective	Yumusakhuylu A.C. et al., 2013 [17]
Wistar albino	10/day for 5 days (i.p.)	50	12	Otoprotective	Erdem T. et al., 2012 [18]
3-months rat					
Wistar rat	100/day for 1 day (i.p.)	100	15	Otoprotective	Simsek G. et al. [35]
Albino–Wistar 3-months rat	20 mg/mL × 0.05 mL/day for 1 days (i.p.)	N/A	15 for 1 day	Otoprotective	Simsek G. et al. [16]
Wistar albino rat	10/day for 10 days (i.p.)	100	16	Ototoxic	Olgun Y. et al., 2014 [20]
SD 8-weeks rat	50/day for 10 days (i.p.)	500	3 for 5 days	Ototoxic	Current study

**Table 2:** Oligonucleotide primer sequences for quantitative reverse transcriptase polymerase chain reaction.

Gene	Primer Sequence (Forward)	Primer Sequence (Reverse)	Annealing Temperature (°C)	Product Size (bp)	RefSeq Number
*IL6*	5′-AGAGACTTCCAGCCAGTTGC-3′	5′-TGAAGTCTCCTCTCCGGACT-3′	60	88	NM_012589.2
*IL1β*	5′-CACCTTCTTTTCCTTCATCTTTG-3′	5′-GTCGTTGCTTGTCTCTCCTTGTA-3′	60	241	NM_031512.2
*NFκB*	5′-TGTCTGCACCTGTTCCAAAGA-3′	5′-TGCCAGGTCTGTGAACACTC-3′	60	143	NM_199267.2
*CYP1A1*	5′-CATCCCCCACAGCACCATAA-3′	5′-TTCGCTTGCCCAAACCAAAG-3′	60	212	NM_012540.2
*GAPDH*	5′-ATTGTTGCCATCAACGACCC-3′	5′-TGACTGTGCCGTTGAACTTG-3′	60	94	NM_017008.4

## Data Availability

The data presented in this study are available on request from the corresponding author.
